# *IL-6* variant is associated with metastasis in breast cancer patients

**DOI:** 10.1371/journal.pone.0181725

**Published:** 2017-07-21

**Authors:** Chike O. Abana, Brian S. Bingham, Ju Hwan Cho, Amy J. Graves, Tatsuki Koyama, Robert T. Pilarski, A. Bapsi Chakravarthy, Fen Xia

**Affiliations:** 1 Department of Radiation Oncology, Vanderbilt University Medical Center, Nashville, Tennessee, United States of America; 2 Department of Radiation Oncology, The Ohio State University Comprehensive Cancer Center, James Cancer Hospital and Solove Research Institute, Columbus, Ohio, United States of America; 3 Department of Urologic Surgery and Center for Quantitative Science, Department of Biostatistics, Vanderbilt University Medical Center, Nashville, Tennessee, United States of America; 4 Center for Quantitative Science, Department of Biostatistics, Vanderbilt University Medical Center, Nashville, Tennessee, United States of America; 5 Department of Internal Medicine, University of Arkansas for Medical Sciences, Little Rock, Arkansas, United States of America; 6 Department of Radiation Oncology, University of Arkansas for Medical Sciences, Little Rock, Arkansas, United States of America; University of North Carolina at Chapel Hill School of Medicine, UNITED STATES

## Abstract

**Introduction:**

Although tumor metastases remain significant drivers of mortality, the genetic factors that increase the risks of metastases are not fully identified. Interleukin 6 (*IL-6*) has emerged as an important factor in breast cancer progression with *IL-6* single nucleotide polymorphism (SNP) variants shown to affect survival. We hypothesized that SNPs of the *IL-6* promoter at rs1800795 in breast cancer patients are associated with distant metastases.

**Methods:**

We performed an initial case-control study using Vanderbilt University Medical Center’s BioVU, a genomic biobank linked to de-identified electronic medical records in the Synthetic Derivative database, to identify germline SNPs that may predict the development of metastatic disease to any site from any solid tumor including breast cancer. We identified a SNP in *IL-6*: rs1800795 to be of significance and evaluated this finding using a separate, matched-pair cohort of breast cancer patients with and without metastases from The Ohio State University Wexner Medical Center.

**Results:**

The initial study suggested that GG relative to CG at rs1800795 (OR 1.52; 95% CI 1.14–2.02; *p* = 0.004) was significantly associated with the development of metastases. This association was also observed in the Ohio State University cohort (OR 2.23; 95% CI 1.06–4.71; *p* = 0.001). There were no significant relationships between rs1800795 status and any patient or tumor characteristics, including estrogen receptor status.

**Conclusions:**

These findings suggest that GG SNP at *IL-6*: rs1800795 may indicate an increased risk of metastasis of primary breast cancer. Further studies in larger population sets are warranted as advanced screening and prophylactic intervention might be employed in GG carriers.

## Introduction

Despite many recent advances in the detection and treatment of breast cancer, the development of metastases remains a significant problem and the main driver of mortality. Indeed, whereas patients with localized breast cancer have a five-year survival rate of 98%, patients with metastatic breast cancer have a five-year survival rate of only 26% [1]. While sobering, such statistics are not surprising given that most cancer-related deaths are caused by metastases rather than primary tumor growth. The development of metastatic disease is a multi-step process which depends on both the tumor and patient characteristics [2]. While there has been an explosion in the knowledge of how tumor-specific gene expression can predict outcomes in the last decade, very little is known about the role that the host genome plays in the behavior of the tumor [3]. Studies have shown primary tumor size, nodal status, time to locoregional failure, histologic grade, and receptor expressions are associated with an increased risk of breast cancer metastases. However, clinicians are still unable to reliably assess an individual patient’s risk of developing metastases as compared to other patients with similar clinical and pathologic features [4].

The mechanism by which primary tumors become metastatic also remains an area of controversy. If tumor-specific mutations are the drivers of metastasis, gene expression profiles of tumor tissue are critically important. On the other hand, if host genome (i.e. germline) mutations also determine metastatic potential, blood samples could be used to develop predictive profiles. This possibility is especially important when considering single nucleotide polymorphisms (SNPs)—the most common genetic variations in humans. SNPs have been hypothesized to be maintained in all cells including blood, stromal, and tumor cells. In this setting, identifying host genome SNPs associated with metastasis holds great promise. Genetic information could be used in combination with clinical and pathologic features to better assess individual-level risk and prescribe increased screening and/or prophylactic therapy. Furthermore, such information could advance our understanding of mechanisms that drive metastasis and create opportunities for better treatment and prevention.

Breast cancer studies suggest that interleukin 6 (*IL-6*) acts as a regulator of estrogen synthesis and aromatase activity, mediates a growth response related to hormone receptor status, and contributes to pro-metastatic processes including epithelial-to-mesenchymal transition, cell invasion, cell migration, and mesenchymal stem cell recruitment [5–7]. In addition, clinical studies have shown increased circulating levels of IL-6 in patients with breast cancer as well as an association between higher circulating levels and more advanced stages of disease including metastasis [8, 9]. As IL-6 serum levels are subject to significant inter- and intra-individual variations, SNPs in the *IL-6* promoter region have recently been studied in relationship to breast cancer progression and survival due to the known influence of promoter region mutations on expression levels of pro-inflammatory cytokines. Among these SNPs, rs1800795 (174 G>C) has emerged as especially important in affecting circulating IL-6 levels, and has been shown to be associated with extranodal extension as well as decreased disease-free and overall survival in breast cancer patients [5, 10, 11]. In general, carriers of the G allele at the rs1800795 (174 G>C) polymorphism have been shown to have higher plasma concentrations of IL-6 [12]. In addition, a recent meta-analysis (13 articles with 3,224 patients) showed that *IL-6* expression is associated with a poor prognosis in breast cancer [13]. Despite the well-established roles of SNPs within immune-related genes in breast cancer development and prognosis [5, 14–16], there are no reports of the effects of *IL-6*: rs1800795 SNPs and breast cancer metastasis. We hypothesized that *IL-6*: rs1800795 SNPs within the host genome of breast cancer patients are associated with a greater risk of developing distant metastases.

To test this hypothesis, we initially evaluated associations between the development of metastases in a large cohort of patients from Vanderbilt University Medical Center (VUMC) with solid tumors using patients with any solid tumor, including breast, with a comprehensive list of 105 biologically relevant SNPs, including *IL-6*: rs1800795. Analysis of these 105 SNP sites revealed a statistically significant association between *IL-6*: rs1800795 and the development of distant metastases. We then repeated the study using a cohort of only breast cancer patients with (cases) or without (controls) metastasis from The Ohio State University Wexner Medical Center that were matched in pairs based on age, race, and time-to-metastasis to further evaluate our finding.

## Material and methods

### Patient selection

Patients from the VUMC cohort were recruited through BioVU, a de-identified biobank of DNA from discarded blood samples of patients; and the Synthetic Derivative, a database linking these DNA samples to their de-identified Electronic Medical Records (EMRs) [17]. Only samples from patients 18 years or older were included. The ninth revision of the International Classification of Diseases (ICD-9) code algorithms for primary cancer diagnoses, including breast (ICD-9 174), and metastatic outcome (ICD-9 196, 197, 198) were used to retrospectively select and classify samples obtained between 2007 and 2012 as either cases or controls [18]. Where available, each patient’s classification was cross-referenced with the tumor registry coding to confirm the primary cancer diagnosis. A total of 277 patients with cancer (35 breast cancer subjects) who had developed distant metastases (cases) and 711 (127 breast cancer subjects) cancer patients who had not developed metastases (controls) were identified for genotyping. The two groups were well balanced in terms of age, race and gender and primary site (breast, lung, colorectal and melanoma). Treatment received was not a factor used for patient selection.

A separate cohort of only breast cancer patients was studied using the Columbus Breast Cancer Tissue Bank of The Ohio State University’s Division of Human Genetics, which continuously enrolls patients in a tissue and clinical registry. Using available prognostic clinical information, host blood-derived genomic DNA samples of subjects with (cases) or without (controls) distant metastases to any site were retrospectively selected. 183 cases and 183 controls were identified and matched as pairs based on clinical and pathologic factors at diagnosis including age category (≤ 40 years old vs. > 40 years old), histopathology, tumor grade, stage and treatment category. Patients were also matched by race, stage at presentation and only control subjects with a minimum follow-up of two years from primary diagnosis were used to match the case subjects who had varying time lapse from primary diagnosis to metastases. For inclusion, patients were required to be adult females with invasive breast cancer and a DNA sample of sufficient quantity and quality for genotyping.

For the samples obtained at VUMC, the standard “consent to treatment” (informed consent) form was modified to include a statement in bold lettering describing the DNA databank concept, and a checkbox for opting out was also placed directly above the signature lines. In addition, a new institutional policy was introduced so that this form is now signed at each hospital admission and outpatient clinic on an annual basis. Patients could also opt out by calling a specific phone number. The sample-handling program was modified to include only subjects with a signed “consent to treatment” document that did not include a tick in the box. The opt-out rate is ∼2.5% of all patients who sign the form [17]. The Vanderbilt BioVU DNA databank represents one of few biobanks with an operational protocol that classifies its samples as non-human subjects for research as determined by the Vanderbilt IRB and the federal Office of Human Research Protections (OHRP). The Code of Federal Regulations, 45 CFR 46.102 (f), defines a “Human Subject” as a living individual about whom an Investigator conducting research obtains either data through intervention or interaction with the individual, or identifiable private information, even in the absence of intervention or interaction, or an individual who is or becomes a participant in research. BioVU accrues DNA samples extracted from blood remaining from routine clinical testing after samples have been retained for 3 days and are scheduled to be discarded. Therefore, there is no intervention or interaction with individuals for research purposes. The resource is linked to a de-identified version of data extracted from an EMR system, called the Synthetic Derivative (SD), in which all personal identifiers have been removed. Thus, there is no identifiable private information attached to the records. The study was approved by the Vanderbilt IRB, and all VUMC subjects opted-in by not checking a box on the standard “consent to treatment” informed consent form. All samples from the Ohio State University cohort were collected prospectively and all patients signed a written, IRB-approved informed consent allowing use of their samples and clinical data for research purposes.

### Samples, genotyping, and quality control

For the VUMC cohort, DNA was extracted from blood samples in the BioVU databank using the Gentra Puregene Blood Kit (Qiagen, Redwood City, CA, USA). Genotyping of 105 biologically relevant SNPs, including *IL-6*: rs1800795 was performed by the Vanderbilt DNA Resources Core using the Sequenom MassARRAY iPLEX platform with primers synthesized with Sequenom’s MassARRAY Designer software (Sequenom, Inc., San Diego, CA, USA). iPLEX products were analyzed via MALDI-TOF mass spectrometry using a MassARRAY Compact mass spectrometer (Sequenom; Bruker Instruments, Billerica, MA, USA) and Sequenom real-time detection software. All isolation and genotyping assays were performed per manufacturer’s recommendations with protocols available upon request. In order to verify genotyping accuracy, marker and sample genotyping efficiencies were examined along with performance of positive and negative controls. Quality control procedures included examination of marker and sample genotyping efficiency, allele frequency calculations and tests of Hardy–Weinberg equilibrium. Exclusion criteria at both sample and SNP levels were <90% call rate.

For the Ohio State University cohort, DNA was extracted from blood samples using an in-house protocol from the Ohio State University Genetics Sample Bank (available upon request). Genotyping was performed using the multiplex SNaPshot assay (Thermo Fisher Scientific, Wilmington, DE, USA) with primers synthesized by Invitrogen (Carlsbad, CA, USA). Snapshot products were analyzed through capillary electrophoresis on a 3130 xl Genetic Analyser (Applied Biosystems, Foster City, CA, USA) using POP-6 polymer and a customized run module, by adding 1 μl sample DNA to 18.5 μl Hi-Di Formamide and 0.5 μl GeneScan-120 LIZ internal size standard (Applied Biosystems, Foster City, CA, USA). Electropherograms were analyzed using the software Genemapper ID version 3.7 software (Applied Biosystems, Foster City, CA, USA) applying custom panel and bin settings available on request. In order to verify genotyping accuracy, we duplicated genotyping of >10% of randomly selected samples and obtained 100% identical results.

For both cohort studies, all genotyping was performed by institutional facilities without access to clinical data and blinded to the study endpoint.

### Statistical analyses

The primary outcome for both studies was the development of distant metastases. For the VUMC cohort, Fisher's exact tests were used to determine the association between the 105 SNPs and metastases. A logistic regression model was fitted for each SNP as a continuous predictor and the Hochberg method was used to control false discovery rate at the 20% level.

For the Ohio State University cohort, univariate analysis of rs1800795 genotype and assessment of the distribution of clinicopathological variables were performed with Pearson’s chi-squared test. Multivariable analysis was then performed with conditional logistic regression models adjusting for known prognostic variables in breast cancer. These factors included age at diagnosis, race (non-Hispanic white, black, or Asian), stage at diagnosis (1, 2, or 3+), grade (1, 2, or 3), and receptor positivity (ER, PR, and HER2). Patients with missing genotype information for rs1800795 were omitted from the analyses.

ANOVA (Analysis of variance) was used for group analyses of allelic variants in both cohorts. Prior data indicated that the probability of exposure among the controls is 0.3 and the correlation coefficient for exposure between matched cases and controls is 0.2. Based on these values, we determined that we would have 80% power to detect true odds ratios for disease of 0.63 or 1.52 in exposed subjects relative to unexposed subjects for the VUMC cohort, and 0.55 or 1.65 in exposed subjects relative to unexposed subjects for the Ohio State University cohort. Type I error probability was set at 5% for both cohort analyses. For all analyses, differences were considered significant when a *p* value <0.05 or false discovery rate <0.20 was achieved, with the exception of the 105 SNP association analyses where a *p* value <0.005 was considered significant. All analyses were performed using R version 3.1 (R Foundation for Statistical Computing, Vienna, Austria).

## Results

Patient characteristics for the VUMC cohort included 277 case patients (with distant metastases) and 711 control patients (no metastasis) are presented in Table 1. There were more males than females in the case group (55.6% vs. 44.4%), while the opposite was observed in the control group (46.4% vs. 53.6%). Age and race distributions between cases and controls were similar. Most patients were non-Hispanic whites and over 51 years old, reflecting the common patient demographic at VUMC. The proportions of breast and other primary sites were 12.6% (n = 35) and 87.4% (n = 242), respectively, in the case group compared to 17.9% (n = 127) and 82.1% (n = 584) in the control group.

**Table 1:** VUMC patient demographics.

Demographics	Case Patients	Control Patients
**Gender**		
Male	154 (55.6%)	330 (46.4%)
Female	123 (44.4%)	381 (53.6%)
**Race**^**a**^		
Non-Hispanic white	239 (89.2%)	617 (90.2%)
Black	28 (10.4%)	60 (8.8%)
Other	1 (0.4%)	7 (1.0%)
**Age**		
<51	33 (11.9%)	95 (13.4%)
51–60	57 (20.6%)	145 (20.4%)
61–70	90 (32.5%)	199 (28.0%)
71–80	60 (21.7%)	153 (21.5%)
81–90	30 (10.8%)	92 (12.9%
>90	7 (2.5%)	27 (3.8%)
**Primary Site**		
Breast	35 (12.6%)	127 (17.9%)
Other	242 (87.4%)	584 (82.1%)
**Total**	277	711

^a^Race information was missing for 9 case patients and 27 control patients.

**Fig 1:**
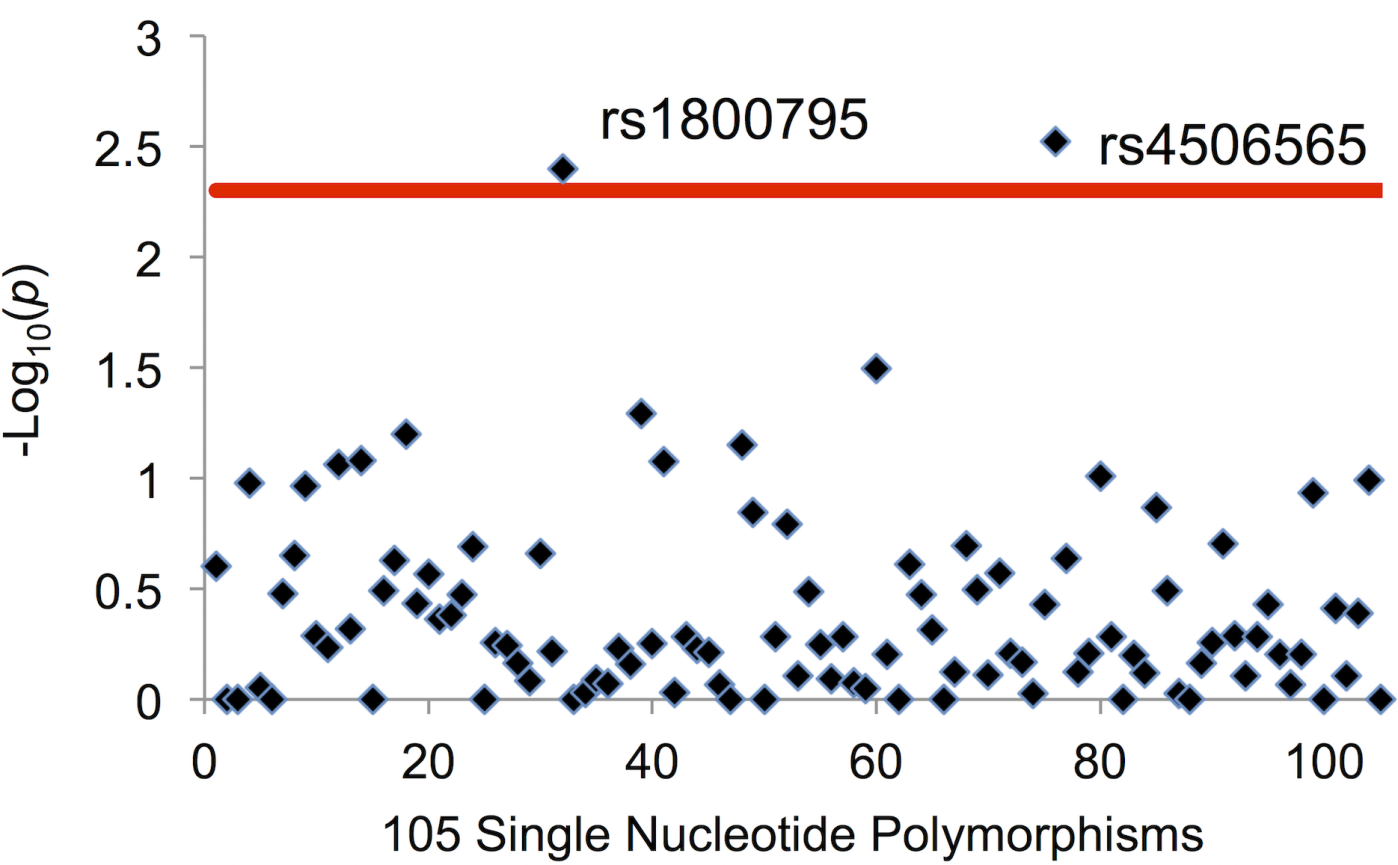
Log_10_(*P*-values) for associations between 105 SNPs and distant metastases in the VUMC study. **-** Threshold for statistically significant association was set at *P* = 0.005 (solid, horizontal red line). *P*-values were obtained using Fisher’s exact test.

**Fig 2:**
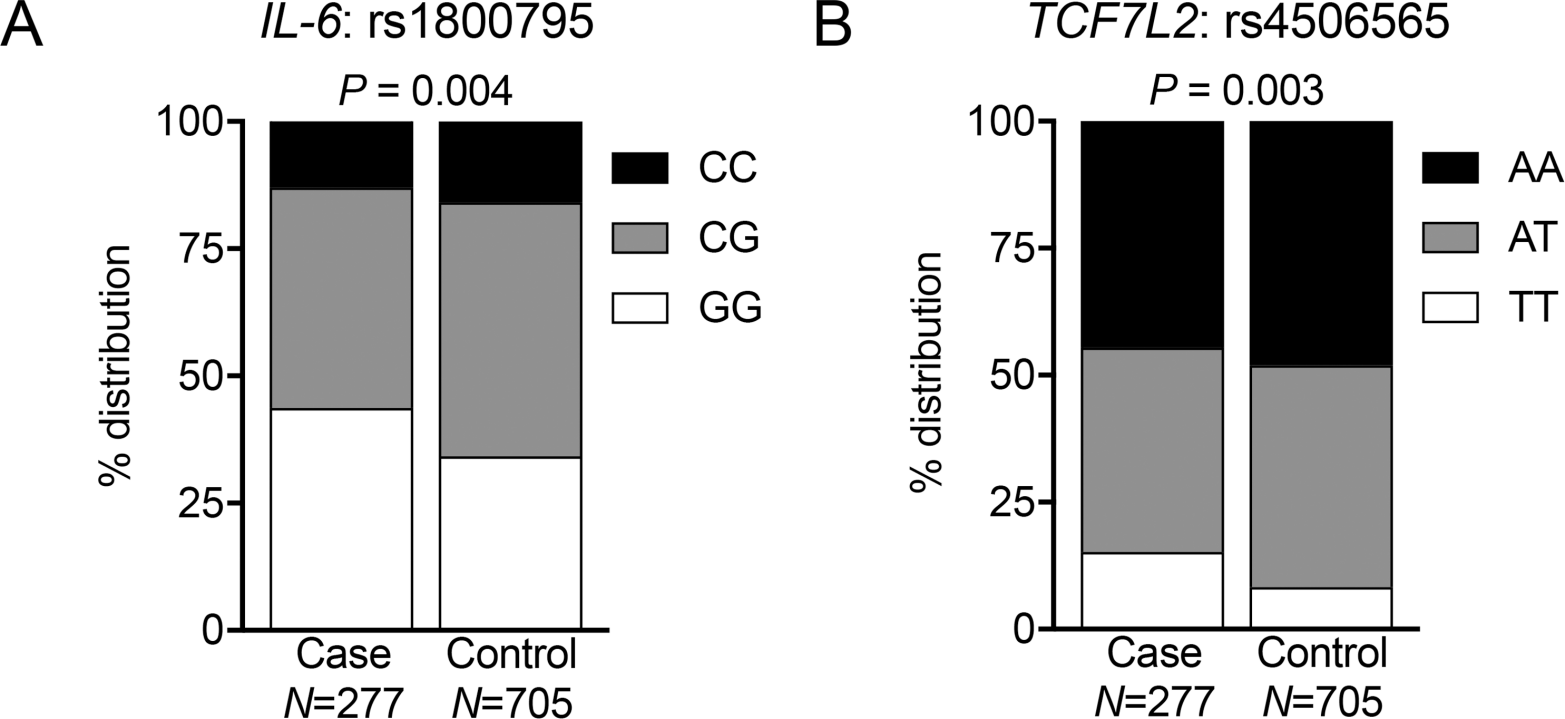
VUMC cohort: rs1800795 and rs4506565 SNPs are associated with distant metastases. Graphs show the proportions of homozygous dominant, heterozygous and homozygous recessive alleles at (A) rs1800795 and (B) rs4506565. Total patient numbers shown represent non-missing values. Six controls had missing rs1800795 genotype information. One case and eight controls had missing rs4506565 genotype information. Statistical analyses show ANOVA test of overall association between SNPs and case or control status.

Analysis of the 105 SNP sites revealed a statistically significant association between rs1800795 SNPs and the development of metastases. Interestingly, a similar association was detected at rs4506565 that is located within an exon of Transcription Factor-7 Like 2, *TCF7L2* (Fig 1). SNP analyses at all other 103 sites did not show any association. Allelic variations analyses indicated that the risk of developing metastases was higher in patients with GG at rs1800795 (OR 1.52; 95% CI 1.15–2.02; *p* = 0.004) and TT at rs4506565 (OR 1.97; 95% CI 1.28–3.00; *p* = 0.003) (Fig 2).

**Fig 3:**
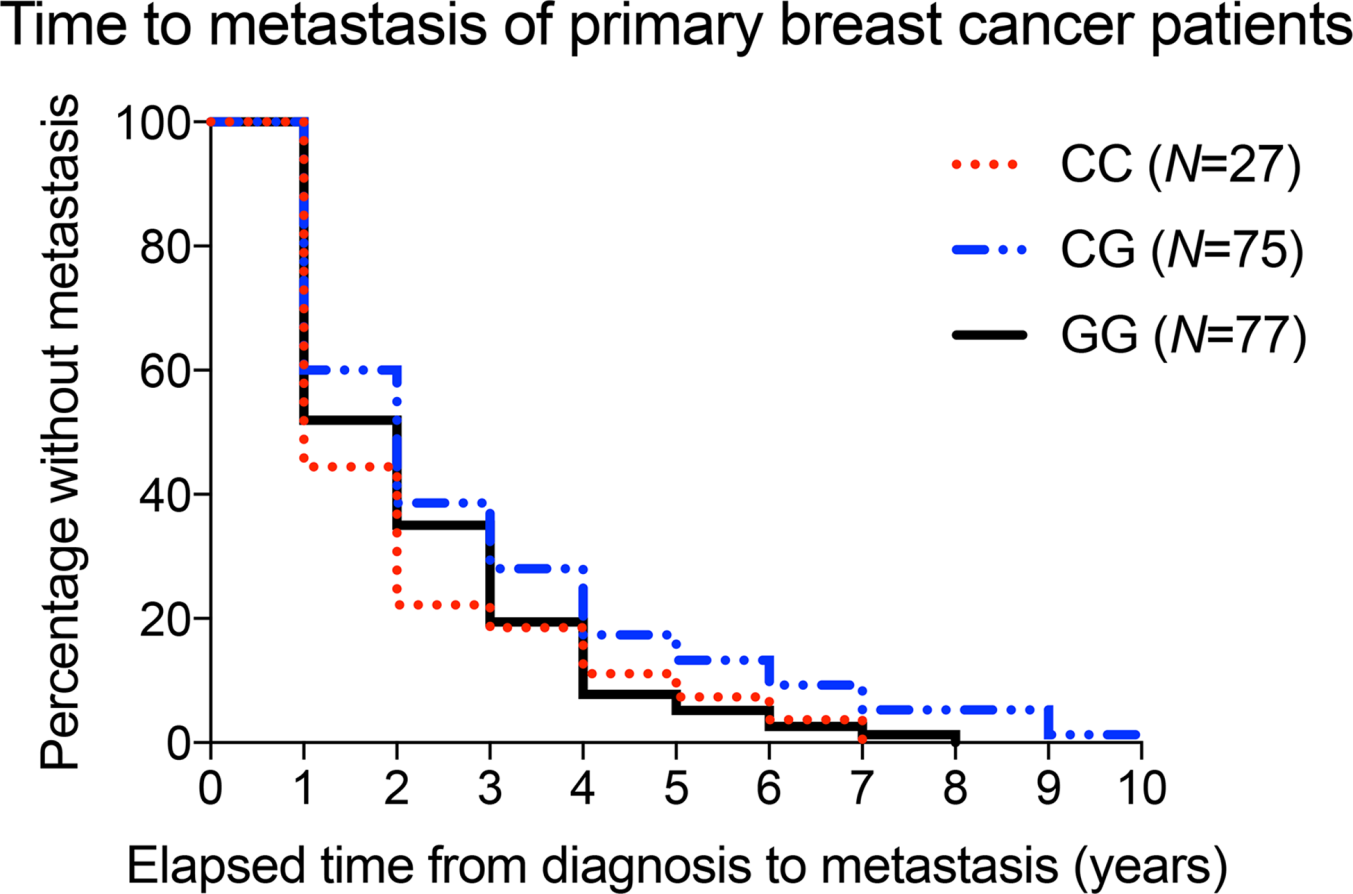
All Ohio State University breast cancer cases develop metastases within ten years from primary diagnosis. Kaplan-Meier curve shows changes in proportion of metastasis-free breast cancer cases as they develop metastasis after primary diagnosis. “CC”: red dotted line; “CG”: blue dotted-solid line, and “GG”: black solid line.

A separate cohort of only breast cancer patients was obtained from Ohio State University. Given the rarity of male breast cancer, male patients were excluded from this analysis. Overall, cases and controls were effectively matched by age, stage, grade and receptor status. Similar to the VUMC cohort, the majority of case and control patients were non-Hispanic white (87.8% and 86.3%) and over 51 years old (56.8% and 55.7%), reflecting the most frequent breast cancer patient demographic at Ohio State University (Table 2). Median follow-up times for the case and control patients were 2.7 and 3.1 years, respectively. The median time-to-metastasis from primary diagnosis for case patients was 1.7 years, and the time-to-metastasis for all patients is presented (Fig 3).

**Table 2:** Ohio State University cohort: Patient and tumor characteristics.

	Case Patients^b^	Control Patients^c^
**Race**		
Non-Hispanic white	157 (86.3%)	158 (87.8%)
Black/Asian	25 (13.7%)	22 (12.2%)
**Age**		
<51	79 (43.2%)	81 (44.3%)
51–60	64 (35.0%)	50 (27.3%)
61–70	24 (13.1%)	31 (16.9%)
71–80	11 (6.0%)	13 (7.1%)
81–90	5 (2.7%)	8 (4.4%)
**Stage at Diagnosis**		
1	21 (12.7%)	28 (16.7%)
2	52 (31.5%)	61 (36.3%)
3+	92 (55.8%)	79 (47.0%)
**Tumor Grade**		
1	16 (9.2%)	23 (12.7%)
2	56 (32.2%)	60 (33.1%)
3	102 (58.6%)	98 (54.1%)
**Estrogen Receptor Status**		
Positive	114 (62.3%)	123 (68.3%)
Negative	69 (37.7%)	57 (31.7%)
**Progesterone Receptor Status**		
Positive	94 (51.4%)	104 (58.1%)
Negative	89 (48.6%)	75 (41.9%)
**HER2/neu Receptor Status**		
Positive	38 (21.0%)	44 (24.6%)
Negative	143 (79.0%)	135 (75.4%)
**Total**^**a**^	183	183

^a^Total patient numbers shown represent total case or control patients identified, including those with missing values.

^b^Race, stage at diagnosis, tumor grade, and HER2/neu receptor status were missing for 3, 18, 9, and 2 case patients, respectively.

^c^Race, stage at diagnosis, tumor grade, estrogen receptor status, progesterone receptor status, and HER2/neu receptor status were missing for 1, 15, 2, 3, 1, and 4 control patients, respectively.

Distributions of racial and age categories of all patients (cases and controls together) by rs1800795 genotypes (CG vs. CC vs. GG) showed statistically significant differences. There were higher and lower prevalence of black/Asian patients among GG and CC carriers, respectively. While there were also significant differences between the genotypes and age categories, this difference is absent when age was analyzed as a continuous variable. We observed no relationship between rs1800795 genotypes and grade or receptor status (Table 3). Based on its observed association in the VUMC cohort, rs4506565 SNP was also analyzed in the Ohio State University cohort, and it had similar breakdowns of patient and tumor characteristics except when age was analyzed as a continuous variable.

**Table 3:** Ohio State University cohort: Patient and tumor characteristics by rs1800795 status.

	CG	CC	GG	*P* Value
**Race**				
Non-Hispanic white	166 (90.7%)	53 (98.1%)	90 (76.3%)	<0.001^a^
Black/Asian	17 (9.3%)	1 (1.9%)	28 (23.7%)	
**Age** (categorized variables)				
<51	77 (41.6%)	22 (40.0%)	56 (47.0%)	0.024^a^
51–60	61 (33.0%)	15 (27.3%)	37 (31.1%)	
61–70	32 (17.3%)	13 (23.6%)	9 (7.6%)	
71–80	11 (5.9%)	5 (9.1%)	8 (6.7%)	
81–90	4 (2.2%)	0 (0.0%)	9 (7.6%)	
**Age** (continuous variable)	53.2±11.7	53.9±12.0	53.3±13.8	0.498^b^
**Tumor Grade**				
1	23 (12.8%)	3 (5.8%)	12 (10.3%)	0.420^a^
2	60 (33.3%)	20 (38.5%)	33 (28.2%)	
3	97 (53.9%)	29 (55.8%)	72 (61.5%)	
**Estrogen Receptor Status**				
Positive	120 (65.6%)	34 (63.0%)	78 (65.5%)	0.934^a^
Negative	63 (34.4%)	20 (37.0%)	41 (34.5%)	
**Progesterone Receptor Status**				
Positive	101 (55.2%)	28 (52.8%)	64 (53.8%)	0.943^a^
Negative	82 (44.8%)	25 (47.2%)	55 (46.2%)	
**HER2/neu Receptor Status**				
Positive	50 (27.3%)	8 (14.8%)	24 (20.7%)	0.117^a^
Negative	133 (72.7%)	46 (85.2%)	92 (79.3%)	
**Total**	**185**	**55**	**119**	

^a^*P* values were determined using Pearson’s χ2 test.

^b^*P* value was determined using Kruskal-Wallis test.

**Fig 4:**
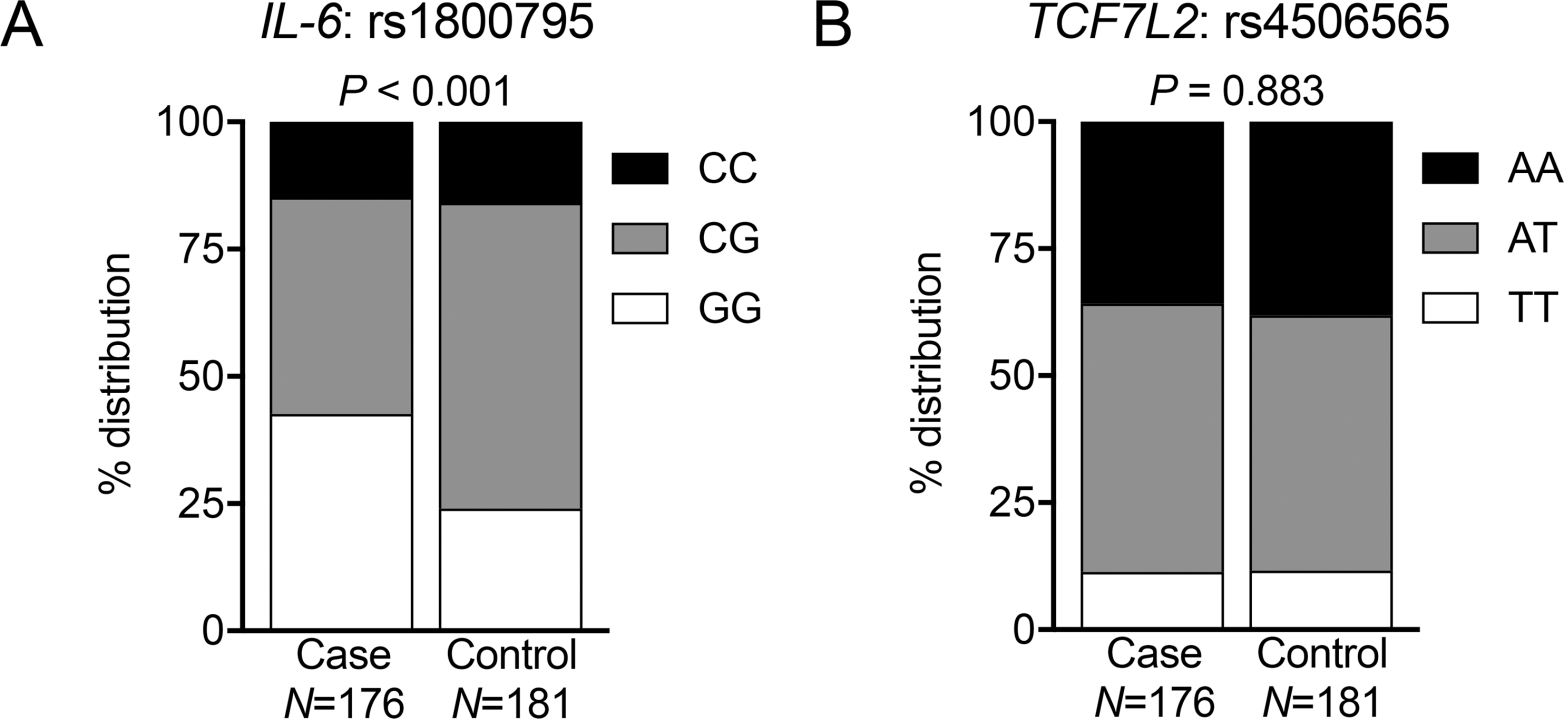
Ohio State University cohort: rs1800795 SNP is associated with distant metastases of primary breast cancer. Graphs show the proportions of homozygous dominant, heterozygous, and homozygous recessive alleles at (A) rs1800795 and (B) rs4506565 from primary breast cancer patients. Total patient numbers shown represent non-missing values. *P* values represent test for overall association between SNPs and case or control status, and were determined using ANOVA.

Conditional logistic regression modeling including all patients with rs1800795 (n = 309) and rs4506565 (n = 307) genotyping was performed. Each variable was assessed for significant association with development of metastasis while controlling for all other variables. Group analyses revealed a significant difference in the distribution of the three variants of rs1800795, but not of rs4506565 (Fig 4).

Using pair-wise analysis, GG relative to CG at rs1800795 was observed to significantly associate with distant metastasis of breast cancer (AOR 2.52; 95% CI 1.39–4.56; *p* = 0.002) (Table 4). In contrast, CC compared to CG showed no significant association. Analyses of patients with rs4506565 genotyping revealed no significant SNP association with breast cancer metastasis with AT vs. AA (AOR 1.65; 95% CI, 0.89–3.06; *p* = 0.112) and TT vs. AA (AOR 2.00; 95% CI, 0.73–5.52; *p* = 0.178). As expected, there was a trend towards statistical significance between late stage (3+) at diagnosis and the development of metastatic disease for rs1800795 (AOR 4.14; 95% CI, 0.92–18.70; *p* = 0.065). Interestingly, HER2/neu positivity was associated with a slightly lower risk of developing metastatic disease for both rs1800795 (AOR 0.45; 95% CI, 0.22–0.94; *p* = 0.033) (Table 4) and rs4506565 (AOR 0.35; 95% CI, 0.16–0.74; *p =* 0.006). The remaining variables including age, race, ER/PR status did not show any association with the development of metastatic disease.

**Table 4:** Ohio State University cohort: Conditional logistic regression model for rs1800795 SNP association with breast cancer metastases.

	Odds ratio	95% CI	*P* Value^a^
rs1800795: CC vs. CG	1.23	0.56–2.70	0.609
rs1800795: GG vs. CG	2.52	1.39–4.56	0.002
Age	0.99	0.96–1.02	0.556
Race: Black/Asian vs. non-Hispanic white	0.77	0.16–3.63	0.742
Stage: 2 vs. 1	0.56	0.13–2.41	0.441
Stage: 3+ vs. 1	4.14	0.92–18.70	0.065
Grade: 2 vs. 1	4.36	0.36–52.58	0.247
Grade: 3 vs. 1	2.68	0.19–37.91	0.466
ER: Positive vs. Negative	0.91	0.37–2.23	0.835
PR: Positive vs. Negative	0.66	0.30–1.49	0.322
HER2: Positive vs. Negative	0.45	0.22–0.94	0.033

^a^Each variable was assessed for significant association with case status (i.e. distant metastasis) while controlling for all other variables.

Using conditional logistic regression model, a subset analysis limited to non-Hispanic whites was performed. Controlling for age, grade and receptor status, rs1800795 remained significantly associated with the development of distant metastases, GG relative to CG at rs1800795 (AOR 2.54; 95% CI 1.32–4.88; *p* = 0.004). In contrast, CC compared to CG showed no significant association (Table 5).

**Table 5:** Ohio State University cohort: Conditional logistic regression model for rs1800795 SNP association with breast cancer metastases among non-Hispanic whites only.

	Odds ratio	95% CI	*P* Value^a^
rs1800795: CC vs. CG	1.06	0.47–2.38	0.897
rs1800795: GG vs. CG	2.55	1.33–4.88	0.005
Age	0.99	0.96–1.03	0.738
Stage: 2 vs. 1	0.33	0.06–1.69	0.183
Stage: 3+ vs. 1	3.55	0.74–17.04	0.114
Grade: 2 vs. 1	1.96	0.16–24.77	0.602
Grade: 3 vs. 1	1.28	0.10–16.79	0.852
ER: Positive vs. Negative	1.07	0.40–2.86	0.899
PR: Positive vs. Negative	0.51	0.21–1.27	0.149
HER2: Positive vs. Negative	0.50	0.22–1.15	0.101

^a^Each variable was assessed for significant association with case status (i.e. distant metastasis) while controlling for all other variables.

## Discussion

To investigate if host genetic variations affect the risk of breast cancer metastasis, we initially evaluated associations between the developments of distant metastases in a large cohort of patients with solid tumors using an unbiased approach of any solid tumor, including breast, with a comprehensive list of 105 biologically relevant SNPs within the host genome. This unbiased approach revealed two SNPs, rs1800795 (within the *IL-6* promoter) and rs4506565 (within an exon of *TCF7L2*), were significantly associated with the development of distant metastases. Specifically, we found higher proportions of GG and TT variants at rs1800795 and rs4506565, respectively, among all metastatic subjects including those with primary breast cancer compared to controls with no metastasis. Using a separate dataset of only breast cancer subjects from Ohio State University, we observed the same finding with rs1800795 but not for rs4506565. To our knowledge, this is the first study to specifically analyze the relationship between host genome or germline rs1800795 SNPs and the metastatic outcomes from primary breast cancer.

Although no previous reports have examined the relationship between rs1800795 and distant cancer metastases, studies have examined the relationship between rs1800795 and prognosis. Given that metastasis is a known driver of cancer mortality, these studies are of particular interest in light of our current findings. For example, GG carriers at rs1800795 have been shown to have decreases in overall survival in gastric cancer [19], in successful treatment rate in Hodgkin’s Disease [20], and in both disease-free survival and overall survival in breast cancer, neuroblastoma and ovarian cancer [5, 21, 22]. The data examining the association of rs1800795 with prognosis and survival in breast cancer patients are especially interesting as they illustrate a relationship potentially influenced by estrogen receptor status and treatment. For example, in a retrospective cohort study of 364 American women with at least 10 positive axillary lymph nodes at diagnosis who underwent adjuvant chemotherapy (Intergroup Trial 0121/E2190/SWOG9061/CALGB 9496), a multicenter trial of high-dose versus standard dose adjuvant chemotherapy, GG was associated with decreased disease-free survival in patients with estrogen receptor positive tumors [5].

Interestingly, in a subsequent prospective cohort study of 634 Swedish women of all stages and treatments, CC was associated with decreased event-free survival (the majority of events representing metastases) in patients with estrogen receptor-negative tumors and among chemotherapy-treated patients irrespective of ER-status [10]. In light of these seemingly contradictory findings, Markkula *et al* hypothesized that the association of GG with increased IL-6 production under normal circumstances is reversed in the presence of inflammatory stimuli such as estrogen receptor negativity, chemotherapy, or radiotherapy (i.e. inflammation causes C-carriers to produce more IL-6). In our analyses of metastases and rs1800795 SNPs, we did not observe a statistically significant difference among patients with estrogen receptor-positive or -negative tumors. While initially surprising, this result could reflect differences in our population from previously studied cohorts including the smaller size or inability to analyze additional variables such as nodal status and treatments received. The difference between this study and previously reported studies may also include differences in the racial composition of the population being studied.

Overall, our results align well with the known role of *IL-6* in pro-metastatic processes and the emerging evidence for associations between rs1800795 polymorphisms and clinical outcomes in patients with breast cancer. Indeed, the possibility that the development of metastasis could be a connecting link between rs1800795 alleles and the decreased survival shown in previous studies is certainly plausible. Importantly, however, the effect of a single SNP on the development of metastasis is likely to be limited and further investigation into other contributory genetic factors is needed.

We discovered an increased prevalence of the rs1800795 GG allele among black and Asian patients. This finding merits additional study in a larger population as a similar observation was made in a previous small study of breast cancer patients [5]. If this observation is found in a larger population, it could potentially contribute to our understanding of the factors responsible for disparities among black patients with breast cancer. In particular, it is interesting to speculate that the racial difference in the prevalence of the GG allele of rs1800795 amongst black patients could potentially explain the clinical observation that more black patients present with brain metastases from breast cancer and also have a worse overall median survival when compared to white patients [23].

The unexpected finding in our study was an association between HER2 receptor positivity and decreased risk of metastases. Given the well-established clinical phenotype of HER2-positive tumors as more prone to relapse and metastasis, the observed association is likely influenced by additional variables such as treatment regimen. In particular, since anti-HER2 therapies are widely available, it is possible that the decreased risk of metastasis that we observed is a result of Trastuzumab therapy, which has been shown to increase both overall and disease-free survival [24]. In the Trastuzumab era, HER2 status has gone from being a negative prognostic factor to a positive predictive factor.

The additional polymorphism associated with metastasis in the VUMC cohort—RS4506565—is located on chromosome 10 within exon 4 of transcription factor 7 like 2 (*TCF7L2*). A transcription factor involved in the transduction of Wnt signaling, TCF7L2 is part of a family of proteins responsible for transactivation of genes needed for embryonic processes such as cell fate determination, proliferation and polarity, as well as processes important in carcinogenesis such as cell cycle progression and apoptosis [25–27]. Indeed, abnormalities in Wnt signaling have been shown to play a role in the development and progression of many cancers including breast cancer. Furthermore, other *TCF7L2* SNPs besides rs4506565 are reported to be associated with increased risks of developing primary prostate, colon, and breast cancer [28–32]. rs4506565 has been investigated only for its role in gestational and type 2 diabetes mellitus [33, 34]. It is possible that the statistically significant association with metastases observed in the initial study for rs4506565 was driven by other solid tumors besides breast, explaining why this association was absent when only breast cancer patients were analyzed in the Ohio State University study. Nevertheless, *TCF7L2*: rs4506565 should be further investigated in future research for potential associations with cancer metastasis.

In conclusion, this is the first study to show an association between the GG allele at rs1800795 within the host genome and the development of metastases from breast cancer. Importantly, there are several weaknesses of this study including its retrospective nature, the lack of racial diversity, the small sample sizes (which precluded further analysis for an association between host genome SNPs and site-specific metastasis), the unavailability of complete clinical information (including staging and treatment details) for all patients, lack of access to tumor and/or tumor stromal tissue (which would have allowed for the assessment of the presence of the SNP in those tissues), and lack of data on locoregional recurrence (which would have allowed for an analysis of SNP association with locoregional vs. distant metastasis). In addition, although the VUMC cohort included both genders, given the rarity of male breast cancer, the Ohio State University cohort was limited to women. Given the possibility of SNP frequency variation between men and women, the difference might cause a disparity between the two data sets. The rate and frequency of metastasis is dependent on tumor type, race and gender. Given the small numbers in the VUMC data set, we were unable to control for all of these variables. A larger well-designed prospective analysis could help address these limitations, and allow for stratification by treatment type and nodal status. If validated, presence of the GG allele at rs1800795 could be combined with other clinical and pathologic factors to identify breast cancer patients at increased risk for metastases for prophylactic screening and therapies.
